# Rubisco Activase Is Also a Multiple Responder to Abiotic Stresses in Rice

**DOI:** 10.1371/journal.pone.0140934

**Published:** 2015-10-19

**Authors:** Yue Chen, Xiao-Man Wang, Li Zhou, Yi He, Dun Wang, Yan-Hua Qi, De-An Jiang

**Affiliations:** State Key Laboratory of Plant Physiology and Biochemistry, College of Life Sciences, Zhejiang University, Hangzhou, 310058, China; Louisiana State University Agricultural Center, UNITED STATES

## Abstract

Ribulose-1,5-bisphosphate carboxylase/oxygenase activase (RCA) is a nuclear gene that encodes a chloroplast protein that plays an important role in photosynthesis. Some reports have indicated that it may play a role in acclimation to different abiotic stresses. In this paper, we analyzed the stress-responsive elements in the 2.0 kb 5’-upstream regions of the RCA gene promoter and the primary, secondary and tertiary structure of the protein. We identified some cis-elements of multiple stress-related components in the RCA promoter. Amino acid and evolution analyses showed that the RCA protein had conserved regions between different species; however, the size and type varied. The secondary structures, binding sites and tertiary structures of the RCA proteins were also different. This might reflect the differences in the transcription and translation levels of the two RCA isoforms during adaptation to different abiotic stresses. Although both the transcription and translation levels of RCA isoforms in the rice leaves increased under various stresses, the large isoform was increased more significantly in the chloroplast stroma and thylakoid. It can be concluded that RCA, especially RCA_L_, is also a multiple responder to abiotic stresses in rice, which provides new insights into RCA functions.

## Introduction

There are differences in the genes and bis-phosphate carboxylase/oxygenase activase (RCA) isoforms among plant species. In many plants, there are two RCA forms: a large 45–46 kD isoform and a small 41–43 kD isoform. Genomic analyses have identified one *RCA* gene in spinach, Arabidopsis, rice and wheat [1–3], in which alternative splicing of the RCA transcript results in two RCA isoforms [2, 4, 5]. Two *RCA* genes encode two RCA isoforms in barley and cotton [5, 6]. In addition to one alternatively spliced RCA gene (rcaA) that produces two RCA isoforms, a second gene (rcaB) encodes only the small isoform of RCA in barley [5]. Although more than three *RCA* genes have been found in tobacco [7] and soybean [8], they only produce the small RCA isoform [9]. The largest difference between the two RCA forms is at the carboxyl terminus [9]. Compared with the small isoform, the large isoform has a carboxy-terminal extension that contains redox-sensitive cysteine (Cys) residues [6, 9, 10]. Both the large and small isoforms can activate Rubisco; however, they exhibit slight differences in their maximal activity [11]. Notably, light modulation of Rubisco in Arabidopsis requires redox regulation of the large isoform via thioredoxin-f [10, 12, 13].

RCA may be important in the acclimation of photosynthesis [14] and the deactivation of Rubisco [15] to high temperature because the isolated spinach RCA is very heat labile [16]. Spinach [17] but not Arabidopsis [18] suggests species specificity for the isoform temperature stability. In rice, the large isoform may play an important role in photosynthetic acclimation to heat stress, whereas the small isoform plays a major role in maintaining the initial activity of Rubisco [19]. Recently, a total of 2,171 salt responsive protein spots have been identified in proteomics studies in 34 plant species [20]. RCA isoforms have been identified among these spots. Proteomic analysis has also identified protein spots that are differentially regulated in response to drought, containing RCA isoforms in barley [21], mulberry [22] and rice [23, 24]. In addition, RCA may respond to heavy metal stress in tobacco plants [25].

Although additional proteomics research have shown that the RCA protein responds to various abiotic stress treatments, it remains unclear whether that response occurs at the promoter level and whether RCA isoforms are regulated by abiotic stresses. Therefore, it is important to distinguish their differences in gene expression and protein content under various conditions. Based on bioinformatics analysis, we predict the environmentally responsive elements and protein structure and determine the change in transcription and translocation of the two isoforms in rice seedlings. The results show that the transcription and translation levels are actually induced by heat, salt, cold, and polyethylene glycol (PEG). The large isoform in both the chloroplast stroma and thylakoid respond more significantly to the stresses than do the small isoform. Therefore, we conclude that RCA in rice is not only the activating enzyme of Rubisco but also a multiple responder to stresses.

## Materials and Methods

### Plant material and stress treatments

As reported by Wang et al. [19], the germinated rice (*Oryza sativa*) seeds were grown in International Rice Research Institute (IRRI) rice nutrient solution and rice seedlings were grown in a greenhouse under a photosynthetic photon flux density (PPFD) of 500 μmol photons m^-2^ s^−1^ controlled at a day/night temperature regimen of 30/22°C. The solution was adjusted to pH of 5.0–5.5 every 2 days and was renewed once in a week. When the seedlings grew to 5-leaf stage, the treatments were conducted. The nutrient solution was added to 200 mM NaCl for salt [26, 27], 20% PEG6000 for drought treatments [28], respectively. The seedlings were incubated at 10°C or 40°C for cold or heat treatments [19, 29, 30]. The leaf blades were harvested after various treatments for 24 h, respectively. The untreated seedlings were used as controls. After sampling, the leaf blades (10 seedlings per treatment) were frozen in liquid nitrogen and stored at -80°C for later use. All treatment were performed in triplicate.

### Isolation of RNA and cDNA preparation

Total RNA was isolated from 100 mg of stress treated and untreated rice leaf blades using the RNAprep Pure plant kit (TransGen Biotech, China). Genomic DNA contamination was removed with DNaseI treatment according to the manufacturer’s instructions. The cDNA was synthesized using 10 μg total RNA for each template. The first strand cDNA was synthesized from 10 μg total RNA using the PrimeScript one step RT-PCR kit (TAKARA, Japan) using the oligo(dT)18 primer according to the manufacturer’s instructions. Three biological replicates were conducted of the stress treatments for RNA and cDNA.

### Cloning and sequence analysis of the *RCA* gene from rice

The rice *RCA*_*L*_ and *RCA*_*S*_ gene were amplified by PCR using F_S_ (5’- ATGGCTGCTGCCTTCTCCTCC -3’), R_S_ (5’- TCAGCTGGATGGCGCAGAACC -3’), F_L_ (5’- ATGGCTGCTGCCTTCTCCT -3’), and R_L_ (5’- TTAAAAGGTGTAAAGGCAGCTGC -3’). In the PCR reactions, we used the first strand cDNAs from Nipponbare rice as a template to amplify the DNA. The full length rice *RCA* genes were cloned into the pMD19T simple vector (TAKARA). The putative recombinant colonies of *E*. *coli* DH5α with the desired amplification were used to isolate the plasmid DNA using a plasmid kit (TianGen). The plasmid DNA was verified for gene insertion by restriction digestion using the KpnI and XbaI enzymes. Restriction digestion confirmed that the putative positive clone contained the RCA insert, and it was then subjected to nucleotide sequencing.

### Analysis of promoters and gene sequence of *RCA_L_* and *RCA_S_*

A BLAST search of the rice genome annotation project database (http://rice.plantbiology.msu.edu/) was used to identify *RCA* genomic DNA sequences, including the 5’ and 3’-UTR, exon and intron sequences [31]. To identify the putative cis-regulatory elements in the promoter regions of *RCA*, we used a 2.0 kb genomic sequence upstream of the translation initiation codon of the *RCA* gene using the PLACE and PlantCARE cis-element database (http://bioinformatics.psb.ugent.be/webtools/plantcare/html/) [32].

### Tertiary structure prediction of rice *RCA*

An online service system, I-TASSER server, was used to determine the structure and function predictions of the RCA protein. The three dimensional structure of RCA was predicted by an online service system iterative threading assembly refinement algorithm (I-TASSER) standalone package (version 1.1). A tertiary model was built based on multiple threading alignments by LOMETS and iterative TASSER assembly simulations [33]. Finally, they were adjusted using the PyMOL software package.

### Validations and structural motif analysis

The backbone conformations of the predicted models were inspected by the Phi/Psi Ramachandran plot obtained from PROCHECK server (http://www.ebi.ac.uk/thornton-srv/databases/pdbsum/Generate.html). The quality of the predicted protein model of RCA was estimated using the qualitative model energy analysis (QMEAN) server (http://swissmodelexpasy.org/qmean/cgi/index.cgi) [34]. The PDBfiles of the modeled RCA protein were subjected to the PDBsum server (http://www.ebi.ac.uk/thornton-srv/databases/pdbsum/Generate.html) for structural motif analysis.

### Analysis of *RCA* proteins and phylogenetic tree

The deduced amino acid sequence of RCA was compared with the respective subunits of monocots, such as Japonica rice, maize, barley, sorghum, and wheat, using multiple amino acid sequence alignment with the ClustalW mega program (http://www.ebi.ac.uk/). The pairwise amino acid sequence identity of RCA among different species and the respective subunits of the plants noted above were calculated using the DNAstar software. The DNAstar aligned amino acid sequences of the RCA isoforms were used to infer the evolutionary relationship using the neighbor-joining method. The evolutionary distances were computed using the Poisson correction method and are in the units of the number of amino acid substitutions per site. These phylogenetic analyses were performed with the MEGA5 software [35] and iTOL online service [36]. In the parameters used for analysis of MEGA5, pairwise alignment are gap opening penalty with 10 and gap extension penalty with 0.1. And multiple alignment are gap opening penalty with 10 and gap extension penalty with 0.2. Besides, the test of phylogeny is according to bootstrap method with 1000 of bootstrap replications. The functional motifs, patterns and biologically significant sites in the RCA amino acid sequence were located with the ExPASy Proteomics Server ScanPro site (http://www.expasy.org/tools/scanprosite/).

### Quantitative real-time PCR

The transcript profile of *RCA* in the leaf blades under different stress conditions was determined using quantitative real time PCR. The qRT-PCR reactions were performed as described previously, using *RCA* primers (F 5'-CGTGACGGGCGTATGGAGAAG-3'; R 5'-GCACGAAGAGCGCCGAAGAA ATC-3') and *RCA*_*S*_ specific primers (F 5'-TTCTGCGC CATCCAGCTGAA-3'; R 5'-CCTCCTCCTCCTATGCA GG-3'). Rice actin and *EF-1α* genes were used as the internal references. The qRT-PCR experiment was repeated three times independently, and in each experiment, three technical replicates were performed for each treatment time point.

### Semi-quantitative RT-PCR

Actin was used as an internal standard (F 5'–TCCATCTTGGCATCTCTCAG–3'; R 5'–GTACCCGCATCAGGCATCTG–3'). The *RCA* gene-specific primers (F 5'–AGCTCGTCGTCCACATCTCCA–3'; R 5'–CTTGATGATGTCTGCCGCCTC–3') were designed for a region that includes the shared region of the *RCA* small isoform (*RCA*_*S*_) and large isoform (*RCA*_*L*_) mRNA. The three biological amplification conditions were used for all genes: 95°C for 5 min; followed by 30 cycles (95°C for 30 s, 60°C for 30 s, 72°C for 60 s), and followed by a final extension at 72°C for 10 min, and each biological replicate was conducted with three technical replicates.

### Rubisco and *RCA* protein quantification by ELISA

The samples were prepared as described by Wang et al. [19]. The presence of the Rubisco subunit and two RCA isoforms in the supernatant and thylakoid were determined by direct ELISA using antibodies produced by our laboratory against the Rubisco large subunit (RLS) or Rubisco small subunit (RSS) and against different isoforms of RCA. The ELISA was measured according to the method described by Wang et al. [19].

### Statistical analysis

The data above were analyzed by one way analysis of variance (ANOVA), and significant differences between the individual means were determined using Duncan’s pairwise comparison test at the 5% and 1% (P<0.05 and P<0.01) confidence levels.

### Co-IP and SDS-PAGE analyses

To investigate the effects of abiotic stresses on the interaction between RCA_L_ and its binding protein, the complex was isolated from leaf extracts with ProFound™ Co-Immunoprecipitation Kit (Pierce, Rockford, IL, USA) according to the manufacturer’s instructions. Antibodies to RCA_L_ were immobilized on a coupling gel to pull down the complex. To exclude non-specific binding between the protein and the coupling gel or between the protein and the antibodies, a non-relevant antibody against the 6×His tag was coupled to the antibody-coupling gel in a parallel assay. The antibody-coupled gels were incubated in the protein extraction supernatant and washed five times with washing buffer (80 mM NaCl, 8 mM sodium phosphate, 2 mM potassium phosphate and 10 mM KCl, pH 7.4), and then the complexes were eluted from the coupling gel. The resulting complexes were separated on 12% SDS—PAGE gel and stained with silver.

### In gel digestion and mass spectrometry

In-gel tryptic digestion of selected spots was performed according to the method described by Wang et al. (2010) [19] with slight modifications. Selected protein spots were excised manually from colloidal Coomassie stained gels and destained with 50% acetonitrile (ACN) in 25 mM ammonium bicarbonate (NH_4_HCO_3_) five times. Gel pieces were then treated with 10 mM DTT in 25 mM NH_4_HCO_3_ and incubated at 56°C for 1 h. After cooling, the DTT solution was immediately replaced with 55 mM iodoacetamide in 25 mM NH_4_HCO_3_ and incubated for 45 min at room temperature (25 ± 2°C), then washed with 25 mM NH_4_HCO_3_ and ACN, dried in a speed vac and rehydrated in 20 μL of 25 mM NH_4_HCO_3_ solution containing 12.5 ng μL^–1^ trypsin (sequencing grade, Promega, WI, USA). After a 10 min incubation on ice, samples were kept at 37°C for overnight digestion. After digestion, the supernatant was collected in fresh eppendorf tubes and the gel pieces were re- extracted by continuous vortexing with 50 μL solution of 1% trifluoroacetic acid (TFA) and ACN (1:1 v/v) for 15 min. Supernatants were pooled together, vacuum dried using a speed vac and then re-suspended in 5 μL of 50% ACN and 1% TFA (1:1 v/v) solution. A matrix-assisted laser desorption/ionization time of flight mass spectrometry (MALDI-TOF MS) analysis was conducted with a MALDI-TOF-TOF mass spectrometer (Bruker Autoflex III martbeam, Bruker Daltonics, Germany). From the above prepared sample, 2 μL was mixed with equal volumes of freshly prepared a-cyano-4- hydroxycinnamic acid (CHCA) matrix in 50% ACN and 1% TFA (1:1 v/v), and finally, 1 μL of the sample was spotted on the target plate.

### MS/MS analysis

Proteins were identified from the obtained monoisotropic peptide masses using the MASCOT search engine (Matrix Science, London, UK; http://www.matrixscience.com) employing Biotools software (Bruker Daltonics, Germany). The following parameters were fixed for database searches: taxonomic category was set to Viridiplantae (green plants), modifications of carbamidomethyl (CS), variable modification of oxidation (M), enzyme trypsin, peptide charge of 1+ and monoisotropic. The similarity search for mass values was performed with existing digests and sequence information from NCBInr and Swiss Prot database. Based on the MASCOT probability analysis (p < 0.05), only significant hits were accepted for protein identification.

## Results

### Prediction analysis of *RCA_L_* and *RCA_S_* promoters

**Fig 1:**
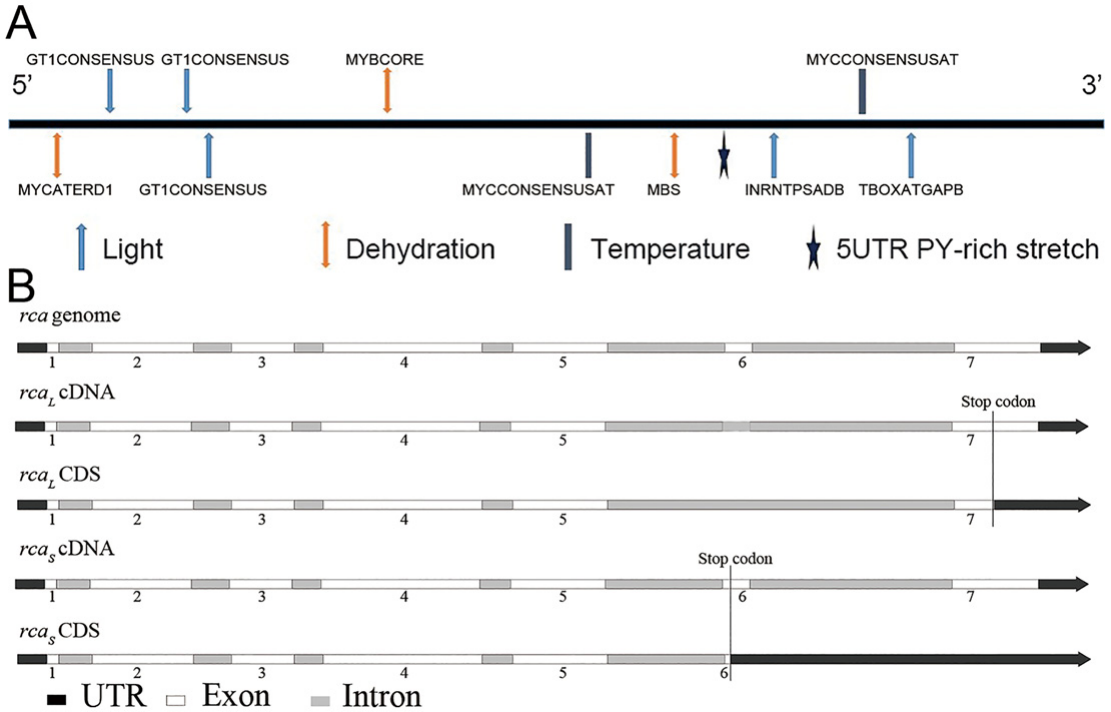
Analysis of RCA’s promoter and genome. (*A*) Analysis of stress-responsive cis-regulatory elements in the 2.0 kb 5’-upstream regions of the RCA gene. (*B*) The schematic representation of genomic organization (exon-intron organization) of the genomic sequence of RCA genes and its alternative splicing. The elements located in the (+) strand are above the lines, whereas those in the (-) strand are indicated below the line.

To understand whether the promoter level of RCA is regulated by abiotic stresses, we analyzed the distribution of regulatory cis-elements in the 2.0 kb upstream promoter region of *RCA*_*L*_ and *RCA*_*S*_ using stress-responsive elements (TC-rich), salt-induced responsive elements (GT-1 motif), heat stress-responsive elements (HSRE), low temperature-responsive elements (LTR) and dehydration-responsive elements (MBS, ACGT). The results in Fig 1A show high frequent distribution of stress responsive elements in the RCA promoter region, including 3 for dehydration and 2 for temperature (Table 1). This indicates that RCA might be regulated by abiotic stresses. In addition, 5 light response elements were also found in the regulatory cis-elements in the 2.0 kb upstream promoter region of *RCA* (Table 1), which confirms that RCA is a light-regulated gene and a stress-regulated gene.

**Table 1:** The cis regulatory elements found in the promoter region of *RCA*.

Function	Total number	Elements name	Copy number
Light	5	GT1CONSENSUS	3
		INRNTPSADB	1
		TBOXATGAPB	1
Dehydration	3	MYCATERD1	1
		MYBCORE	1
		MBS	1
Temperature	2	MYCCONSENSUSAT	2
high transcription levels	1	5UTR PY-rich stretch	1

### Difference in genomic organization and protein structure of *RCA_L_* and *RCA_S_*

The complete coding sequences of *RCA*_*L*_ and *RCA*_*S*_ were amplified using PCR with first-strand cDNA templates prepared from total RNA. Similar to a previous report [2], the alignment of the genomic sequences of *RCA*_*L*_ and *RCA*_*S*_ with their respective cDNA sequences identified six exons (36, 303, 187, 473, 283 and 119 bp) and five introns (99, 113, 85, 92 and 1032 bp) in *RCA*_*L*_ and six exons (36, 303, 187, 473 283 and 19 bp) and five introns (99, 113, 85, 92 and 347 bp) in *RCA*_*S*_. The number of exons and introns was similar between *RCA*_*L*_ and *RCA*_*S*_, but the size of *RCA*_*L*_ was larger. (Fig 1B)

**Fig 2:**
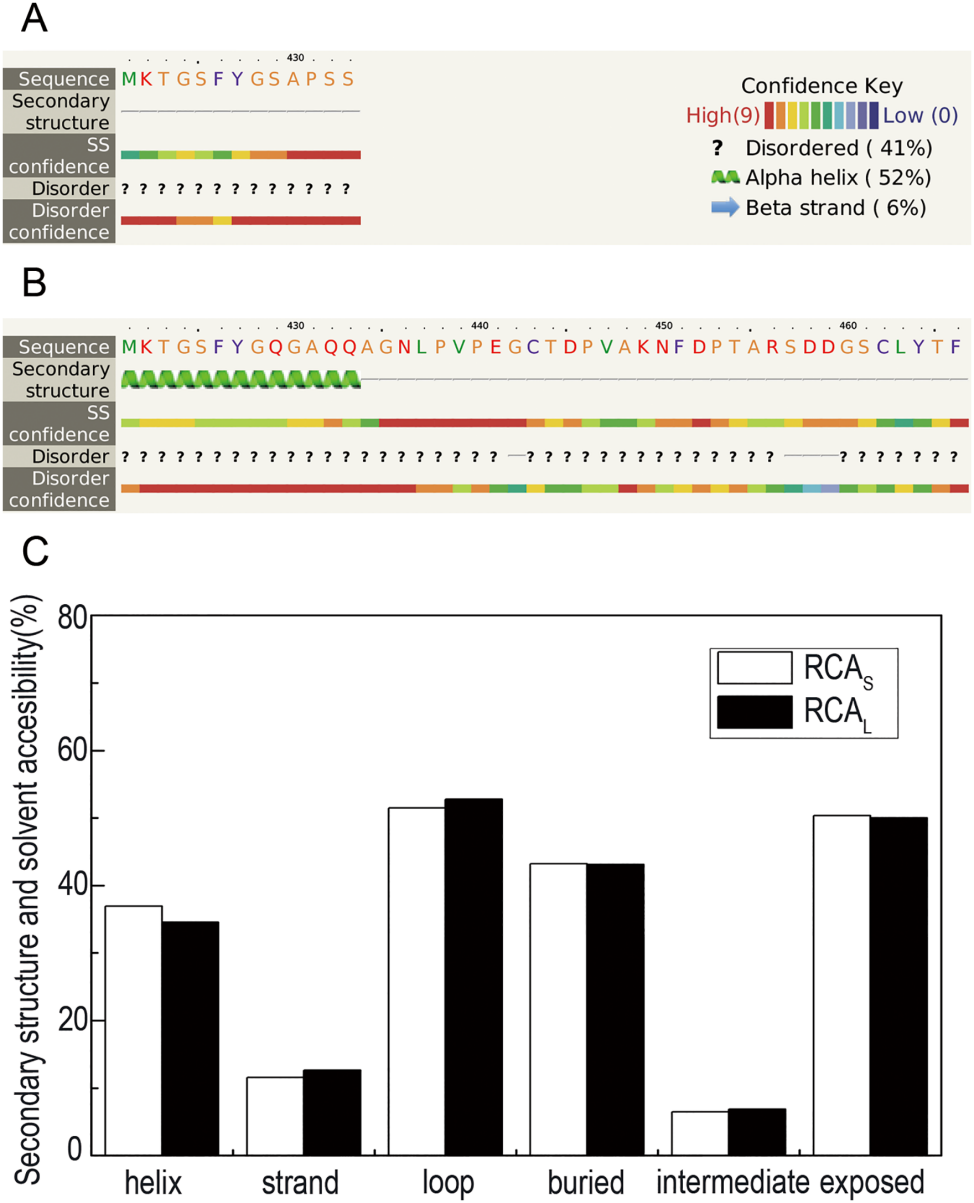
The differences in secondary structure between RCA_L_ and RCA_S_. (*A*) The secondary structure and disorder confidence of RCA_S_. (*B*) The secondary structure and disorder confidence of RCA_L_. (*C*) The (*B*) The quantitative analysis of the secondary structure of RCA.

The analysis showed that there were 5 different consecutive AAs (amino acids) between RCA_S_ and RCA_L_, except that RCA_L_ had an additional 33 AAs at the C-terminal compared with the RCA_S_ in rice (Fig 2A and 2B). Therefore, RCA_L_ had 2 additional strands (436–438,462–465) and 3 additional exposed regions compared with RCA_S_ based on the REPROF rec and PROF Acc server websites. The quantification of these differences is shown in Fig 2C. *RCA*_*L*_ has more strands, intermediate and loop regions, and has fewer helix and exposed regions. In addition, there are more disorder regions (22 of 38 AA) in RCA_L_ because of the different AAs according to the PROF bval, Ucon and MD servers. More disorder regions mean that RCA_L_ has more regions that are partially or wholly unstructured and do not fold into a stable state in three-dimension structure.

**Fig 3:**
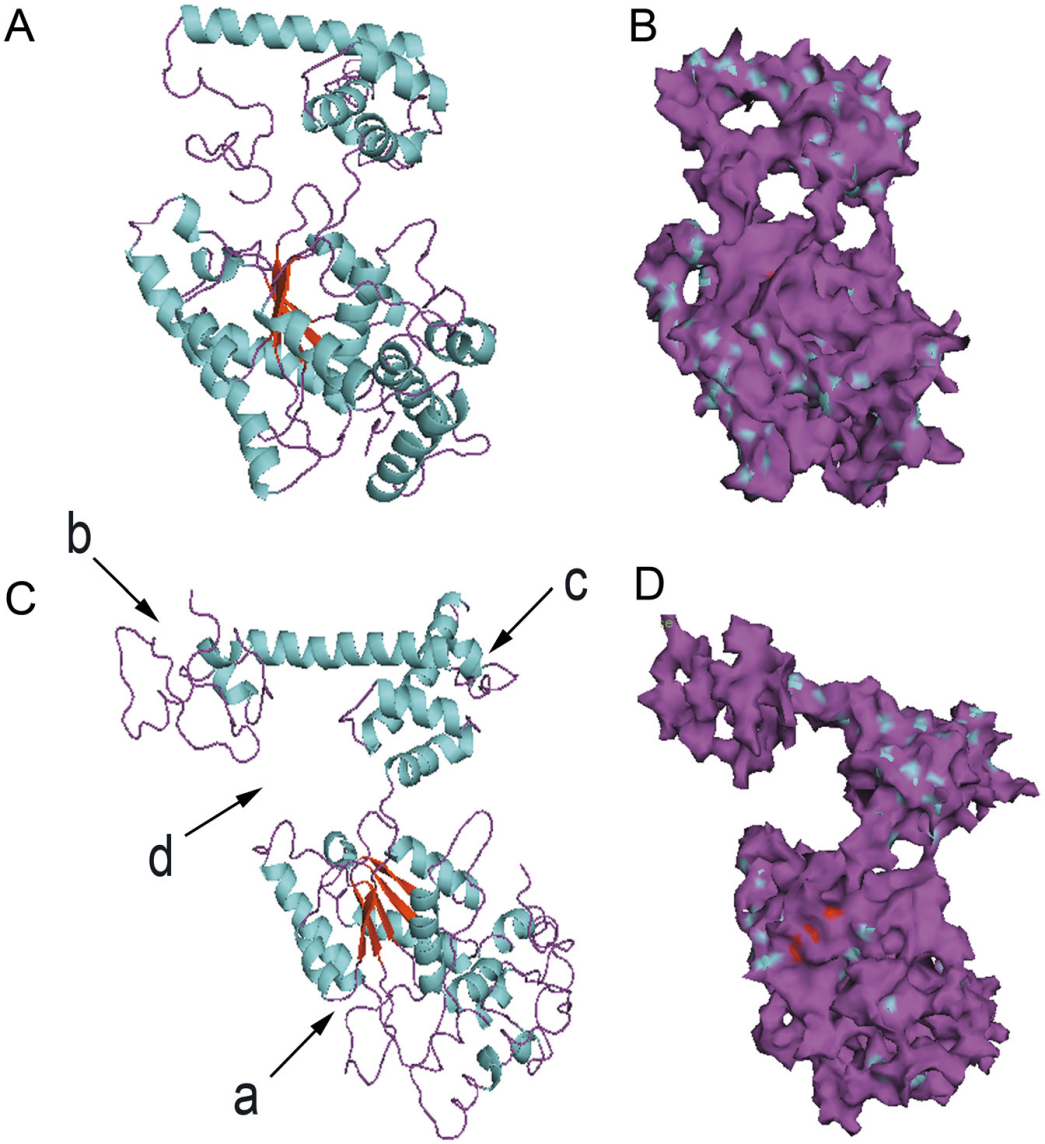
The differences in tertiary structure between RCA_L_ and RCA_S_. (*A*) and (*B*) Prediction of the 3D structure of RCA_S_ and RCA_L_ based on the PDB structure. (*C*) and (*D*) Prediction of the 3D structure of RCA_S_ and RCA_L_ based on the PDB structure. The labels (a, b, c, d) indicate the different domains between RCA_L_ and RCA_S_.

The structure of various homogeneous domains was searched from the PDB bank, and the tertiary structure of RCA was identified and analyzed using the Phyre^2^ server (http://www.sbg.bio.ic.ac.uk/phyre2/html/page.cgi?id=index) and PyMOL software (Fig 3). The tertiary structure of RCA_L_ was different from RCA_S_ because of the 38 additional AAs at the C-terminal. Within RCA_L_ and RCA_S_, the AAA^+^ domain (label a) was conserved. However, the C-terminal (label b) in RCA_L_ was different from that in RCA_S_. This difference resulted in an additional gap (label d). Furthermore, the domain near the C-terminal (label c) was also different. The difference between both isoforms implied that they had different functions. In particular, the additional gap in RCA_L_ means that it might have more functions associated with protein-binding.

### Prediction of active sites and interaction with *RCA_L_* and *RCA_S_* structure

**Fig 4:**
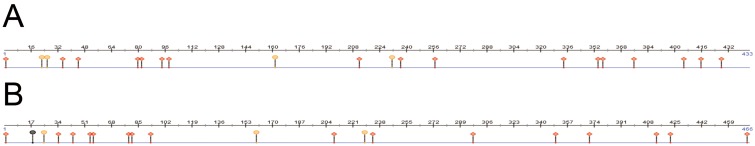
The predicted DNA/RNA-binding sites and protein binding sites of RCA_L_ and RCA_S_. (*A*) The predicted RCA_S_ sites. (*B*) The predicted RCA_L_ sites. Dots indicate the DNA/RNA-binding sites, and the rhombus indicates the protein binding sites.

The binding sites of the target protein were predicted using the ISIS methods [37] and the “Improvement of DNA- and RNA-Protein Binding Prediction” method. A total of 21 active binding sites were predicted in RCA_S_. Among them, only 4 DNA/RNA-binding sites are shown in Fig 4A. In RCA_L_, the active binding sites were reduced to 20 (Fig 4B), which were significantly altered near the C-terminus (Fig 4). These results suggested that the two RCA isoforms bonded to different proteins and had different functions under varying conditions.

### *RCA_L_* and *RCA_S_* phylogenetic tree

**Fig 5:**
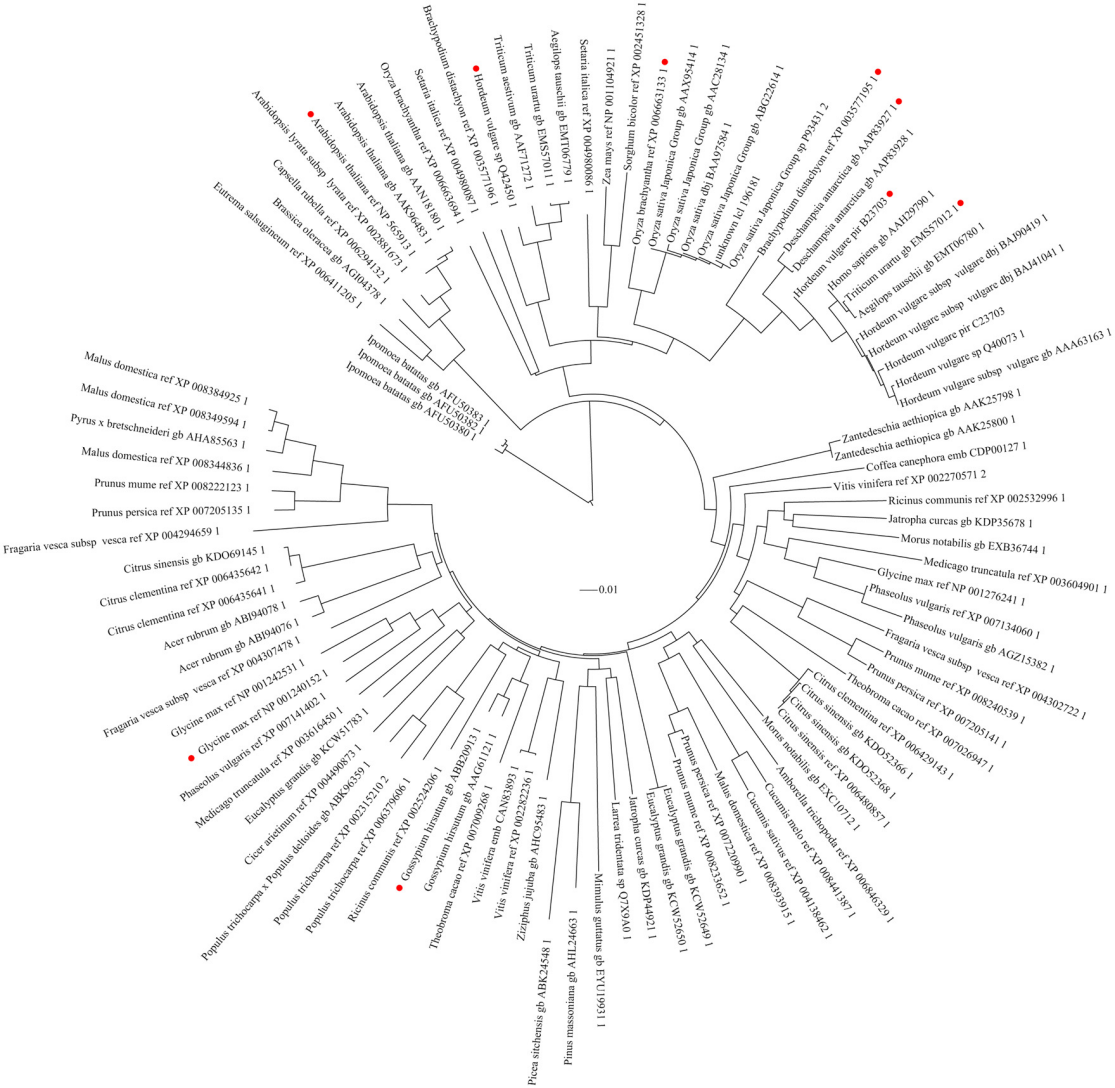
A rooted phylogenetic tree constructed using MeGa5 and iTOL showing evolutionary relatedness of RCA in plants. Red dots indicate RCA_L_ with conservative different amino acids between RCA_L_ and RCA_S_. The bootstrap values from 1000 replicates are shown for selected branches.

**Fig 6:**
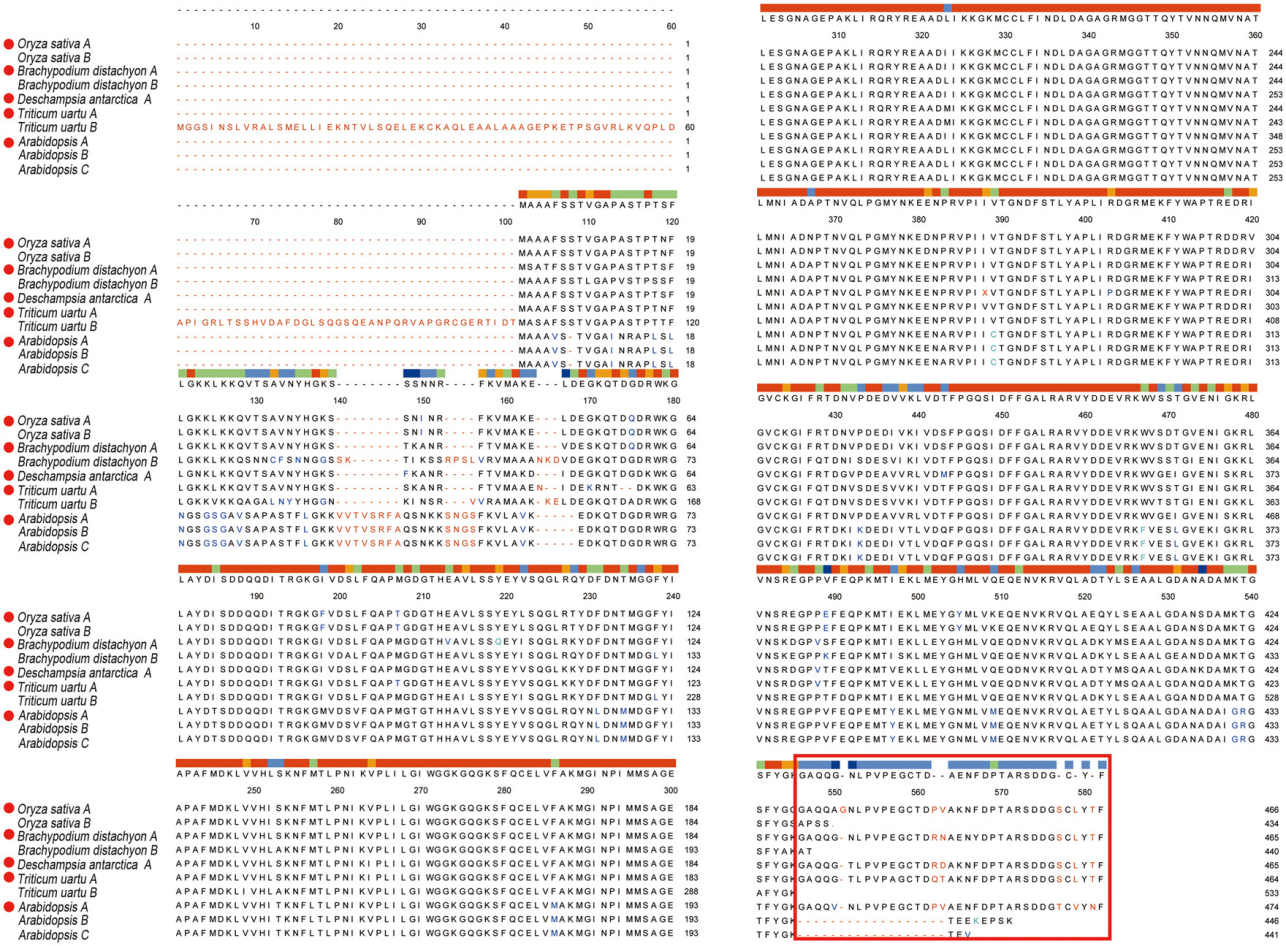
Multiple sequence alignment of RCA protein sequences in several species. The red dot indicates an RCA_L_ in different species, and the red box indicates RCA_L_-specific differential fragments that are more than the RCA_S_-specific ones. Different amino acid sequences of the RCA protein aligned using clustal Omega.

The phylogenetic tree constructed for RCA clustered all plants together (Fig 5) at the amino acid level. The hypothetical proteins within the first 100 similar proteins by blastP of RCA_L_ protein were also considered in this study. This figure is made using the complete sequence, in which the conservative between two enzymes is higher than in the diversity sequence, so the RCA_L_ of different species is dispersed in the figure. The amino acid sequence alignments of the RCA isoforms with their corresponding subunits from *Oryza sativa*, *Brachypodium distachyo*, *Deschampsia antarctica*, *Triticum uartu*, and Arabidopsis are listed in Fig 6 and Table 2. The RCA_L_ and RCA_S_ of rice shared 99% identity with each other, and the C-terminal AAs in RCA_L_ were also conserved according to the red box RCA_L_-specific differential fragments of acid sequence alignment result (Fig 6). Moreover, the observed phylogenetic relatedness suggested that the evolution of RCA in rice was similar to closely related grass family members, such as *Brachypodium* and *Deschampsia*. These results indicated that the structure of RCA was conservative, whereas the different amino acids between RCA_L_ and RCA_S_ were also conservative within many species. It suggests that the different function of RCA_L_ may be the result of evolution.

**Table 2:** Pairwise amino acid sequence identity of *RCA_L_* and *RCA_S_* among different plants.

	Percent Identity											
Divergence		1	2	3	4	5	6	7	8	9	10	
	1		98.6	89.5	85.8	89.5	89.6	87.2	81.8	80.2	80.9	*Oryza sativa japonica* group A
	2	1.4		88.7	86.1	88.5	88.9	87.2	80.5	81.5	81.5	*Oryza sativa japonica* group B
	3	11.4	12.3		85.8	93.3	92.4	86.2	79	77.9	78.6	*Brachypodium distachyon* A
	4	15.7	15.4	15.7		85.2	84.2	91	78.8	79.2	79.2	*Brachypodium distachyon* B
	5	11.4	12.5	7	16.6		96.1	86.5	79.7	78.6	79.3	*Deschampsia antarctica* A
	6	11.2	12.1	8	17.8	4		85.9	78.3	77.3	78	*Theobroma cacao* A
	7	14.1	14.1	15.2	9.6	14.9	15.6		79.4	79.4	79.4	*Theobroma cacao* B
	8	20.9	22.6	24.7	25	23.8	25.7	24.2		98.7	99.5	*Arabidopsis thaliana* A
	9	23.1	21.4	26.3	24.5	25.3	27.1	24.2	1.4		99.8	*Arabidopsis thaliana* B
	10	22.1	21.4	25.3	24.5	24.3	26.1	24.2	0.5	0.2		*Arabidopsis thaliana* C

### Expression of *RCA* isoforms under various abiotic stresses

**Fig 7:**
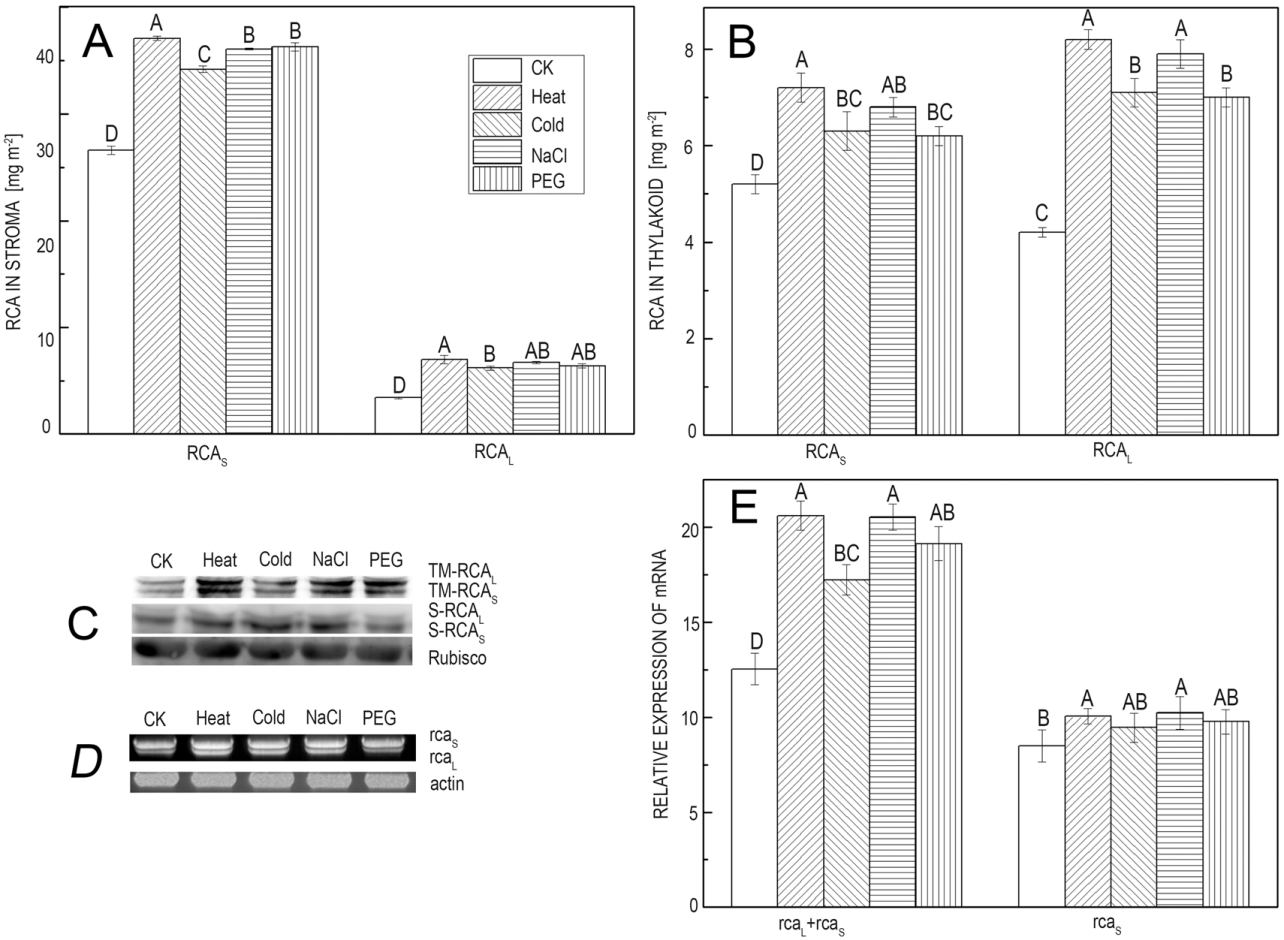
Protein and mRNA *RCA* levels under different abiotic stress conditions. (*A*) ELISA analyses showing the protein content of RCA_L_ and RCA_S_ in the soluble fraction under abiotic stress. (*B*) ELISA analyses showing the protein content of RCA_L_ and RCA_S_ in the thylakoid fraction under abiotic stress. (*C*) Western blotting results regarding RCA_L_ and RCA_S_ in the soluble and thylakoid fractions under different abiotic stress conditions. (*D*) Semi-RT-PCR analyses showing the transcript profile of RCA_L_ and RCA_S_ mRNA under different abiotic stress conditions. (*E*) Quantitative real-time PCR (qRT-PCR) showing the relative content of RCA_L_ and RCA_S_. Actin and EF-1α were selected as reference genes. The statistical analysis was performed using one-way analysis of variance (ANOVA) with Duncan’s pairwise comparison test to compare the mean differences in the fold change expression of RCA during different treatments with respect to the control. The data are expressed as the mean ± SD of three independent experiments, and each experiment consisted of three technical replicates. A capital letter indicates values that differ significantly at P< 0.01 according to Duncan’s pairwise comparison test.

The changes in the two RCA isoform proteins in rice leaves under various abiotic stresses were assessed using western blot and ELISA based on specific monoclonal antibodies against both isoforms and RCA_L_ only. The ELISA results showed that for soluble RCA in the chloroplast stroma in rice, RCA_S_ was 4~5 times higher than RCA_L_, and the two RCA isoforms were significantly up-regulated for 24 hours of stress (Fig 7A). As the bound RCA separated from the thylakoid membrane, both isoforms were up-regulated under all stressed conditions, and RCA_L_ increased more than RCA_S_, particularly for the heat and NaCl treatments (Fig 7B). Western blotting also confirmed the same results (Fig 7C). These indicated that RCA_L_ was more highly induced under abiotic stresses.

To determine whether the protein isoforms of RCA were relevant to mRNA accumulation, the quantitative changes in the two RCA isoform mRNAs in rice leaves during the same abiotic stresses were determined using semi-RT-PCR (Fig 7D) and qRT-PCR (Fig 7E). All of the stresses significantly increased the total *RCA* mRNA (*RCA*_*L+S*_) (Fig 7D and 7E). Because the quantification of *RCA*_*L*_ expression was impossible in our experiment, we deduced that the levels of *RCA*_*L*_ mRNA were increased by several fold under all of the treatment conditions because the increase in the *RCA*_*S*_ mRNA was not as much as that of total *RCA* mRNA (*RCA*_*L+S*_) (Fig 7E). These findings were consistent with the RCA protein in response to abiotic stresses and indicated that the increased RCA protein levels were attributed to the promoted transcription of *RCA*_*L*_ mRNA under abiotic stresses.

## Discussion

### *RCA* may be involved in the response to many abiotic stresses

RCA, a nuclear-encoded chloroplast protein, catalyzes Rubisco activation during photosynthesis by removing inhibitors from the catalytic sites of Rubisco *in vivo* [9, 38, 39]. However, some studies have reported that RCA quickly responds to stress conditions and treatment with plant hormone, which is quite different from the functions of Rubisco activation of RCA accepted by most researchers. Komatsu et al. [40] reported that a gibberellin-binding protein in rice is homologous to RCA, and Sharma and Komatsu [41] suggested that RCA is associated with Ca^2+^-dependent protein kinases in gibberellin signaling. Dejimenez et al. [42] first reported that 45 kD RCA_L_ is induced at high temperature (45°C) and disappears at normal temperature. They suggested that RCA is a possible new member of the molecular chaperone family. These studies suggested additional roles for RCA beyond Rubisco regulation [9]. Furthermore, RCA is an AAA^+^ family protein with diverse functions. The enhanced thermostability of RCA improves photosynthesis and growth under moderate heat stress [43]. RCA may be an important factor in determining the response of boreal plants to global warming based on studies of the dominant species in the boreal forests of North America [44]. Proteomics studies have shown that RCA accumulates under drought stress in barley [21], mulberry [22], and rice [23, 24]. RCA also responds to heavy metal stress in the tobacco plant [25]. Our results indicated that many stress responsive elements exist in the regulatory cis-elements in the 2.0 kb upstream promoter region of RCA (Fig 1), including dehydration and temperature stress responses. In addition, the light response elements may be related to the activation of Rubisco during photosynthesis. This not only supports the results described above but also implies the function of the RCA response to stress conditions at the genome level.

### *RCA_L_* mainly responds to stress through its protein binding ability

Although RCA is an AAA^+^ family protein with diverse functions, it remains unknown whether the two isoforms have different functions *in vivo*. Heat treatment and transgenic rice show that RCA_L_ may play an important role in photosynthetic acclimation to moderate heat stress *in vivo*, whereas RCA_S_ plays a main role in maintaining the initial activity of Rubisco under normal conditions [19]. Our results confirmed that RCA_L_ increases more significantly than does RCA_S_ at both the protein and mRNA levels in response to various abiotic stresses (Fig 7). Simultaneously, the proportions of RCA_L_ and RCA_S_ in the thylakoid increase more (Fig 7B and 7C). The ratio of RCA_L_ and RCA_S_ is approximately 1:4 in the stroma, whereas it is 1:1 in the thylakoid. This is not consistent with the observation that RCA catalyzes Rubisco activation during photosynthetic carbon assimilation because many reports show that the photosynthetic rate is reduced during various stresses [14, 19, 21, 22].

The two RCA isoforms have the same genomic organization and 97% AA sequence homology, except for RCA_L_, which has an additional 38 AAs behind the C-terminal in rice. RCA_L_ may provide the same RNA/DNA-binding regions, similar protein-binding regions and the same AAA^+^ conserved domain for the stereo-structure. Prediction of the three-dimensional structure of RCA using the I-TASSER server and PyMOL software suggests that the RCA_L_ C-terminal domain changes significantly (Fig 2) and that there is a gap within the structure of RCA_L_ (Fig 3). The protein binding function may be due to this gap. Furthermore, the RCA binding proteins are significantly increased under heat stress, suggesting that the increased RCA_L_ may influence the protein binding ability of RCA. This may also explain why the light modulation of RCA is controlled by the redox state of thioredoxin-f via the critical cysteine residues of the C-terminal extension in the larger RCA isoform [10, 39].

In most plants, two RCA isoforms are the products of the same *RCA* mRNA by alternative splicing [2, 4, 5]. The sequence alignment result of the RCA in different species also verifies that the extra AAs in RCA_L_ are highly conserved. In normal photosynthesis, *RCA* mRNA is edited to form more RCA_S_ that are used for the activation of Rubisco and then for CO_2_ assimilation [39]. This is mainly due to the simple structure of RCA_S_ and its relatively high catalytic efficiency regarding Rubisco. RCA_L_ is relatively conserved and shows very low content under normal photosynthesis [39]. However, under stress conditions, its content is significantly increased to protect other functional proteins from damage under stress conditions. The variety of RCA_L_’s binding proteins in the complex increases in stress-acclimated leaves. Considering these findings, experiments to determine whether specific proteins could combine with RCA_L_ in rice under many abiotic stresses need to be performed. We employed co-immunoprecipitation (Co-IP) (S1 Fig) and mass spectrometry methods for this investigation. These results suggest that plants could balance the relationship between photosynthesis and stress tolerance by adjustment of the RCA_S_ and RCA_L_ content under stress conditions.

## Supporting Information

S1 Fig The difference in RCA_L_’s binding protein under different abiotic stress conditions. (TIFF)

S1 File The stress-responsive cis-regulatory elements in the 2.0 kb 5’-upstream regions of the RCA gene. (PDF)

## Abbreviations

AAs: amino acids
AAA+: ATPase associated with a variety of cellular activities
ELISA: enzyme-linked immune sorbent assay
RCA: Rubisco activase
RCA_L_: RCA large isoform
RCA_S_: RCA small isoform
Rubisco: RuBP carboxylase/oxygenase
