# Supplementary figures and images for: Rubisco Activase Is Also a Multiple Responder to Abiotic Stresses in Rice
Source: PLoS One. 2015 Oct 19;10(10):e0140934. doi: 10.1371/journal.pone.0140934 (PMC4610672; doi:10.1371/journal.pone.0140934)

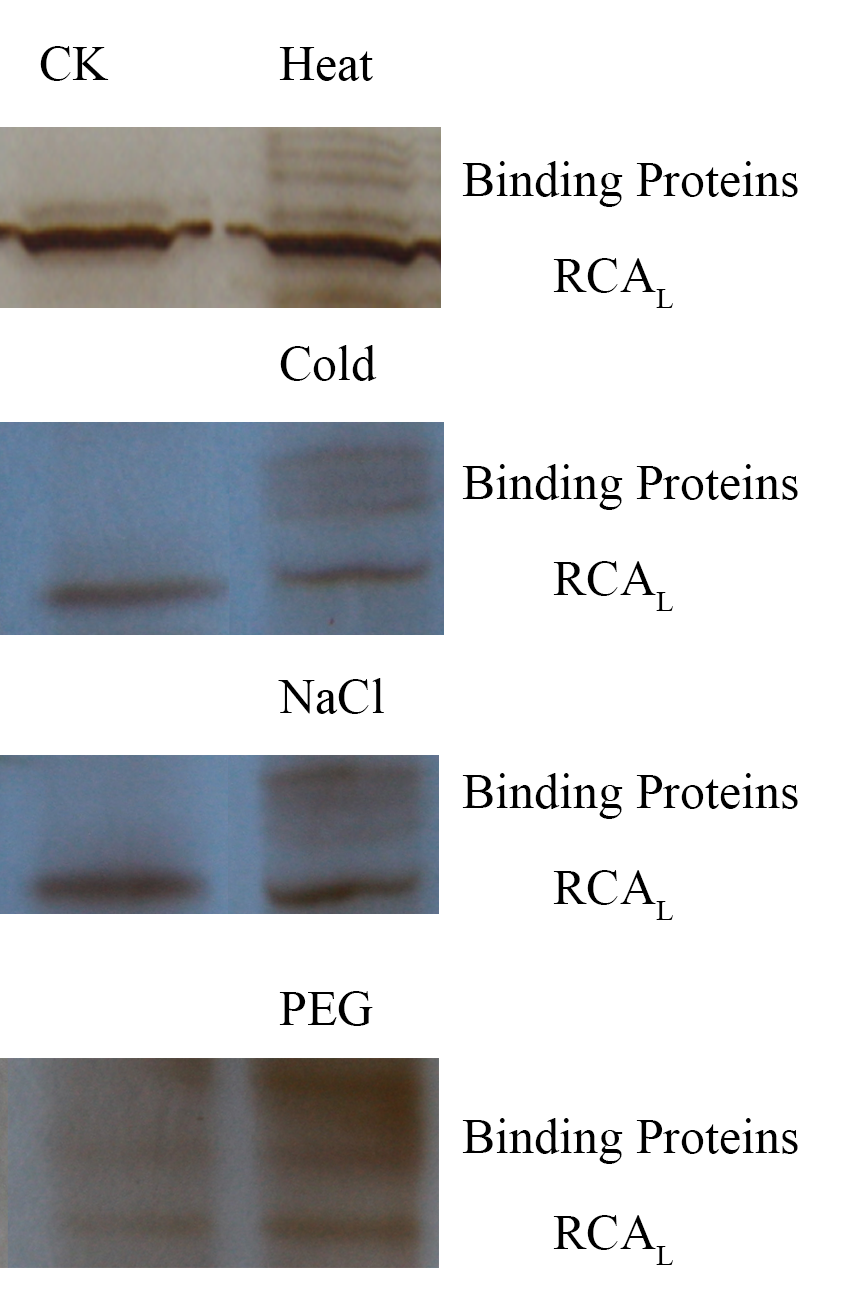

Supplement: S1 Fig — (TIFF) [file pone.0140934.s001.tiff]
