# Supplementary material for: Rubisco Activase Is Also a Multiple Responder to Abiotic Stresses in Rice
Source: PLoS One. 2015 Oct 19;10(10):e0140934. doi: 10.1371/journal.pone.0140934 (PMC4610672; doi:10.1371/journal.pone.0140934)
Supplement: S1 File — (PDF) [file pone.0140934.s002.pdf]

+ ACTTGATTAA TGAATATATA TATATATATA TATATGAACA CCATCGATAT GCTTCCTTGT ACTTCGCACC CTATATAGCC
- TGAACTAATT ACTTATATAT ATATATATAT ATATACTTGT GGTAGCTATA CGAAGGAACA TGAAGCGTGG GATATATCGG

+ TGTACCCTTA ACAAAGAGGA ACGTTTAATT TGTCACTACT GCACCCAAAT TAACTAGTAT TATTTAAATT AATCGTATGA
- ACATGGGAAT TGTTTCTCCT TGCAAATTAA ACAGTGATGA CGTGGGTTTA ATTGATCATA ATAAATTTAA TTAGCATACT

+ GAATTTTTAA AGTATAGTAC ATACTAAAAT TTTACACTAC AAATTTTAAT ACCTCGAAGT ATGTGATATG TGGTGAAATT
- CTTAAAAATT TCATATCATG TATGATTTTA AAATGTGATG TTTAAAATTA TGGAGCTTCA TACACTATAC ACCACTTTAA

+ ACACTTGTCA TATACTAATA CATTATTAGT ATTTTTGCAC ATAATCCAAT CGTTTGTCAC ATAGTTCATT AATTATCCAC
- TGTGAACAGT ATATGATTAT GTAATAATCA TAAAAACGTG TATTAGGTTA GCAAACAGTG TATCAAGTAA TTAATAGGTG

+ TAGTGTACTA GCAGTACTGT ATGCATATGC AACGATACGT ACGATCTCTG GATACTGTGG ATGTGCCATC ATATCGCTGA
- ATCACATGAT CGTCATGACA TACGTATACG TTGCTATGCA TGCTAGAGAC CTATGACACC TACACGGTAG TATAGCGACT

+ TTGATATATA CACATGCTGA CTATATACGG GGATATCCCC TAGTTATTTG CATGTCATTC AAATGATTAT AAAAAAATTT
- AACTATATAT GTGTACGACT GATATATGCC CCTATAGGGG ATCAATAAAC GTACAGTAAG TTTACTAATA TTTTTTTAAA
MYCATERD1
GT1CONSENSUS
+ GAAAAAAAAT TGAGAAGGTC TATTAACATG TGATATAACA CTCCACAAAT ATACAAGTTC AAGTTCAACT TCTACAAATT
- CTTTTTTTTA ACTCTTCCAG ATAATTGTAC ACTATATTGT GAGGTGTTTA TATGTTCAAG TTCAAGTTGA AGATGTTTAA

+ GTAATGAAAA ATACAAATTA AACAACAGCT AGTTAATGTA TATTCAGAGT TAAATTTGTT TTTTTCGTTA CGAGATGTCG
- CATTACTTTT TATGTTTAAT TTGTTGTCGA TCAATTACAT ATAAGTCTCA ATTTAAACAA AAAAAGCAAT GCTCTACAGC

GT1CONSENSUS
+ AAGTTGAATT TTTATTTGCA TGTTTGTAGA GTGATATATC ACATGTTAAT ATATCTTCTC AATTTTTTTC ATAACCACTT
- TTCAACTTAA AAATAAACGT ACAAACATCT CACTATATAG TGTACAATTA TATAGAAGAG TTAAAAAAAG TATTGGTGAA

+ GAGTGACATG CAATCAACGA GGGAATATTT CCTTGAGGGA TCCAAATCCA CTTCCCTATA TATGGATGTA TTCTCAACTT
- CTCACTGTAC GTTAGTTGCT CCCTTATAAA GGAACTCCCT AGGTTTAGGT GAAGGGATAT ATACCTACAT AAGAGTTGAA
GT1CONSENSUS

+ GCGGGTTGCA TTTGTTACTA CAGATGCATG CTATATCAAA GTTCCTGTGC TATTCATCAT GTTGCACGAT AACAATTAGC
- CGCCCAACGT AAACAATGAT GTCTACGTAC GATATAGTTT CAAGGACACG ATAAGTAGTA CAACGTGCTA TTGTTAATCG

```
+ GTATCACTGT AAATCTGTAA TGATGCACAA GTTGGGGAAT GTTATCCAAT TCCAATATGT GCGCTAGTAC TTGCCTAGTG
- CATAGTGACA TTTAGACATT ACTACGTGTT CAACCCCTTA CAATAGGTTA AGGTTATACA CGCGATCATG AACGGATCAC

        MYBCORE
+ AGATGACTGT TATTTTCCAA TGTACTGGAG ACTCGACCTT ACAAGTGTCG TTACCAATCA CTTGCCTCCA TCTCAAACGT
- TCTACTGACA ATAAAAGGTT ACATGACCTC TGAGCTGGAA TGTTCACAGC AATGGTTAGT GAACGGAGGT AGAGTTTGCA

+ CTTAGTAGGG ACAAGCTAGC TAGGAGAAAT TTCACATGAC TTGCATAAAA GATTACTACT GACATGACAT GAGCAAACAA
- GAATCATCCC TGTTCGATCG ATCCTCTTTA AAGTGTACTG AACGTATTTT CTAATGATGA CTGTACTGTA CTCGTTTGTT

+ TTAATTTTCA AAAGTTACGA AAAGCAATAT AGAGGCTTAG GAGGAACGAA AGCTATGAAC CACAAATAAA TTCATCTTAA
- AATTAAAAGT TTTCAATGCT TTTCGTTATA TCTCCGAATC CTCCTTGCTT TCGATACTTG GTGTTTATTT AAGTAGAATT

+ GGCACTGCTG CAGATTTAGC TGCCTCCGAT TGATGCTTCA CCAAAAAACA ATATCAACAG CAGTGCAAAA TTAGAATTTT
- CCGTGACGAC GTCTAAATCG ACGGAGGCTA ACTACGAAGT GGTTTTTTGT TATAGTTGTC GTCACGTTTT AATCTTAAAA

+ TGTATTTTTG TGGTAACGGA AACCATCAAA GGGGAAAAAA CGTACAATGC TTATGTTGTA TGTTAAGAGA AGTTTGTGTG
- ACATAAAAAC ACCATTGCCT TTGGTAGTTT CCCCTTTTTT GCATGTTACG AATACAACAT ACAATTCTCT TCAAACACAC

+ GTGCCAAATG ACAGTCCTAG CCTGATGGTT ATCGAGAAAG CAGAATATGT GCAGGTAGCA GAGCAAAATA TTTGTGGTAG
- CACGGTTTAC TGTCAGGATC GGACTACCAA TAGCTCTTTC GTCTTATACA CGTCCATCGT CTCGTTTTAT AAACACCATC
      MYCCONSENSUSAT

+ TCCAACTAGA ATACAATTTG CATGCCATGC CTCATCCAAG AAGCCGGGCA ACGAGAGGCA GCAAAAGGCT TTTCTGTGGT
- AGGTTGATCT TATGTTAAAC GTACGGTACG GAGTAGGTTC TTCGGCCCGT TGCTCTCCGT CGTTTTCCGA AAAGACACCA

+ GATGCAAAAT GAAGAGGTTA TGTAGTAGCT GAGCTGATGA AGCAACTGGT CGCTAGCTGC CGGCCGGGAG ACGAATGTGA
- CTACGTTTTA CTTCTCCAAT ACATCATCGA CTCGACTACT TCGTTGACCA GCGATCGACG GCCGGCCCTC TGCTTACACT
                                            MBS

+ GGCAAGGAAA GAAAAGAAAA AACAGAGAGA AAGAGTTGAT CAGAAATGGG TGAATTCTGT GGTGAGGAAA GGTCAAGGAA
- CCGTTCCTTT CTTTTCTTTT TTGTCTCTCT TTCTCAACTA GTCTTTACCC ACTTAAGACA CCACTCCTTT CCAGTTCCTT
           5UTR PY-rich stretch                                                   INRNTPSADB

+ CTGAAGCCAA GAGATCCTTC CTACCTACAC TAATACAATA TACTCCTAAC TCGCTCACAG ACTCCGATCC AGGTCCAAGT
- GACTTCGGTT CTCTAGGAAG GATGGATGTG ATTATGTTAT ATGAGGATTG AGCGAGTGTC TGAGGCTAGG TCCAGGTTCA
```

+ CATGCTATGC TGTGGATCGG CCGGCCGAGA TTGCGCCACG TGTGCAGAAC CCAATCTTCA GCGTGTGGCC TGTGGAGGAT
- GTACGATACG ACACCTAGCC GGCCGGCTCT AACGCGGTGC ACACGTCTTG GGTTAGAAGT CGCACACCGG ACACCTCCTA
MYCCONSENSUSAT

+ CTGGAAGCTG ATCCACAGGG ACGAGTGTGT GCCTCTCACA GCCTCCAACT TCCATGGCGA CGTCCAATTC TATTGTATTA
- GACCTTCGAC TAGGTGTCCC TGCTCACACA CGGAGAGTGT CGGAGGTTGA AGGTACCGCT GCAGGTTAAG ATAACATAAT

+ TTTAAGGCCT ACCGCAGCTC GGCCTCTACA CTTTGAGCAG CAGCGGCCGG CCATCATCAG TGATCCTCTA CAATCATCGA
- AAATTCCGGA TGGCGTCGAG CCGGAGATGT GAAACTCGTC GTCGCCGGCC GGTAGTAGTC ACTAGGAGAT GTTAGTAGCT
TBOXATGAPB

+ CTTTCAGCAA ATTAAG
- GAAAGTCGTT TAATTC

***** Light ***** Dehydration ***** Temperature ***** 5UTR PY-rich stretch
